# Giant left atrium in a patient with Marfan syndrome

**DOI:** 10.1093/ehjcr/ytae350

**Published:** 2024-07-12

**Authors:** Felipe Israel López-Trejo, Elias Noel Andrade-Cuellar, Edil Rosalio Argueta Machado

**Affiliations:** Clinical Cardiology, National Medical Center ‘November 20th’, ISSSTE, Av. Felix Cuevas #540, Col. Del Valle Del. Benito Juarez, C.P. 03100 Mexico City, Mexico; Clinical Cardiology, National Medical Center ‘November 20th’, ISSSTE, Av. Felix Cuevas #540, Col. Del Valle Del. Benito Juarez, C.P. 03100 Mexico City, Mexico; Clinical Cardiology, National Medical Center ‘November 20th’, ISSSTE, Av. Felix Cuevas #540, Col. Del Valle Del. Benito Juarez, C.P. 03100 Mexico City, Mexico

A 41-year-old man with Marfan syndrome, complicated by retinal detachment, heart failure with mildly reduced ejection fraction, and atrial fibrillation for 1 year, presented to the cardiology clinic due to worsening dyspnoea. He had no significant family history. On physical examination, vital signs were normal. He had an ectomorphic, dolichocephalic physique with no jugular venous distension. Heart auscultation revealed irregular heart sounds and a mesosystolic murmur at the mitral focus. The Walker–Murdoch wrist sign was positive. Laboratory analysis showed no abnormalities. The electrocardiogram indicated atrial fibrillation and left ventricular hypertrophy. Coronary angiography revealed slow coronary flow. The trans-oesophageal echocardiogram showed left atrial dilation with an anteroposterior diameter of 81.8 mm, volume of 793 mL, and volume index of 425 mL/m^2^. Additionally, there was posterior leaflet prolapse of the mitral valve resulting in severe regurgitation classified as Carpentier type IIA. Magnetic resonance imaging (MRI) measurements showed the left atrium measured 113 × 83 mm, with an area of 122.2 cm^2^ (*Figure 1* and Supplementary material).

**Figure 1:**
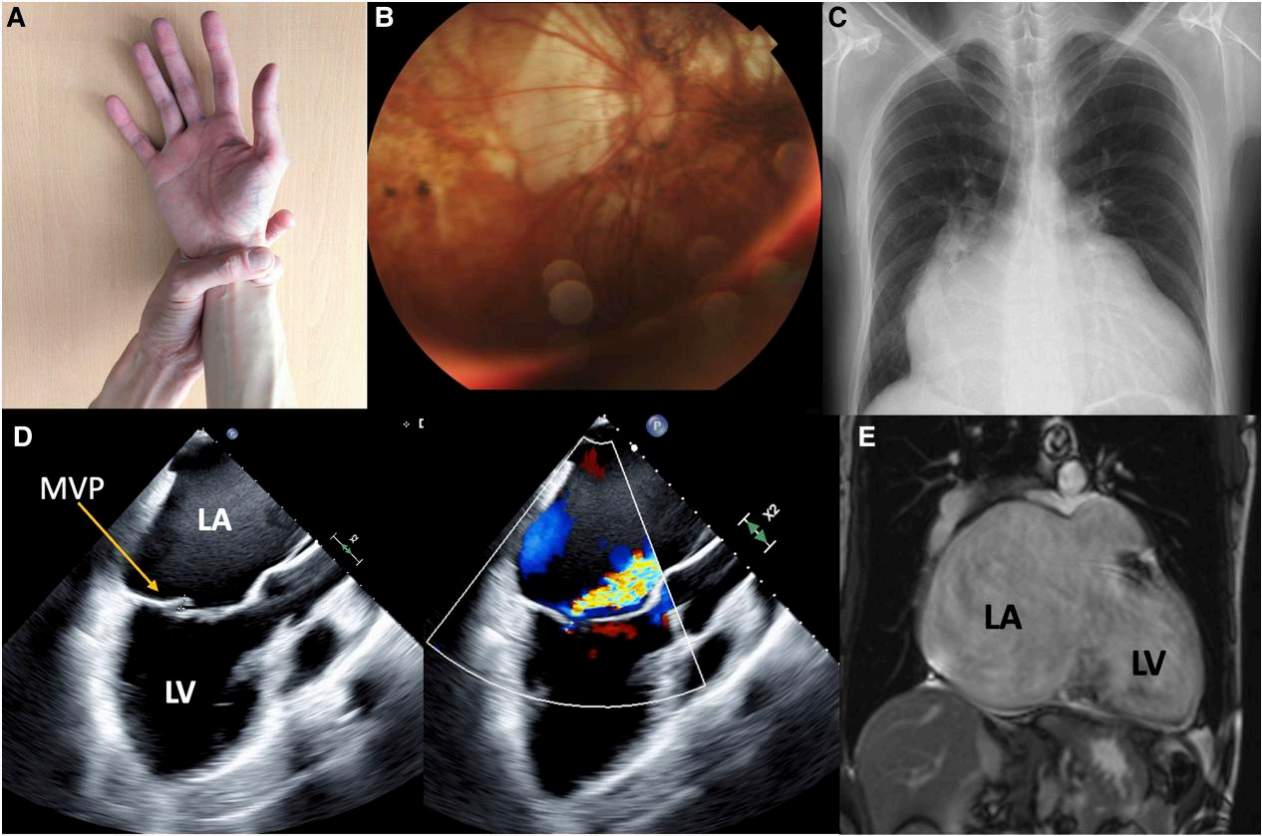
Giant left atrium in a patient with Marfan syndrome. (*A*) Walker–Murdoch wrist sign; (*B*) the funduscopic examination showing myopic choroidosis with chorioretinal atrophy; (*C*) chest X-ray showing cardiomegaly (cardiothoracic ratio 0.81); (*D*) trans-oesophageal echocardiogram (left side) showing giant left atrium comparing left ventricle, with posterior valve prolapse of the mitral valve (arrow), with colour Doppler showing severe regurgitation (right side); (*E*) cardiac magnetic resonance imaging showing giant left atrium and left ventricle in a coronal axis. LA, left atrium; LV, left ventricle; MVP, mitral valve prolapse.

Due to a decline in functional status attributed to severe mitral regurgitation, the case was reviewed by the heart team, who approved mitral valve replacement surgery. Currently, the patient is classified as New York Heart Association (NYHA) functional class II, with the heart rate controlled by a beta-blocker. The incidence of a giant left atrium is 0.3–0.6%, predominantly secondary to rheumatic mitral disease in up to 92% of cases, with non-rheumatic aetiologies being rare.^1^ A retrospective study associated Marfan syndrome with left atrial dilation, suggesting primary atrial involvement in this syndrome.^2^

## Supplementary Material

ytae350_Supplementary_Data

## Data Availability

The data underlying this article are available in the article and in its online supplementary material.

## Supplementary material

**Funding:** The authors have reported that they have no relationships relevant to the contents of this paper to disclose.
